# Three Naturally-Occurring Psychedelics and Their Significance in the Treatment of Mental Health Disorders

**DOI:** 10.3389/fphar.2022.927984

**Published:** 2022-06-28

**Authors:** Nataliya Vorobyeva, Alena A. Kozlova

**Affiliations:** ^1^ Hive Bio Life Sciences Ltd., London, United Kingdom; ^2^ Department of Psychiatry and Psychotherapy, University Hospital Carl Gustav Carus, Technische Universität Dresden, Dresden, Germany

**Keywords:** serotonergic psychedelics, mental health, psilocybin, ibogaine, DMT

## Abstract

Classical psychedelics represent a family of psychoactive substances with structural similarities to serotonin and affinity for serotonin receptors. A growing number of studies have found that psychedelics can be effective in treating various psychiatric conditions, including post-traumatic stress disorder, major depressive disorder, anxiety, and substance use disorders. Mental health disorders are extremely prevalent in the general population constituting a major problem for the public health. There are a wide variety of interventions for mental health disorders, including pharmacological therapies and psychotherapies, however, treatment resistance still remains a particular challenge in this field, and relapse rates are also quite high. In recent years, psychedelics have become one of the promising new tools for the treatment of mental health disorders. In this review, we will discuss the three classic serotonergic naturally occurring psychedelics, psilocybin, ibogaine, and N, N-dimethyltryptamine, focusing on their pharmacological properties and clinical potential. The purpose of this article is to provide a focused review of the most relevant research into the therapeutic potential of these substances and their possible integration as alternative or adjuvant options to existing pharmacological and psychological therapies.

## Introduction

Psychedelics are a broadly-defined class of substances, characteristic psychological effects of which include but not limited to altered sense of self and affective processing, unusual sensory phenomena, such as, for example, synaesthesia. Despite generally broad affinity profiles it has been shown that potency of classical psychedelics is proportional to their ability to bind to serotonin (5-HT) type 2 receptors (Glennon et al., 1984; Sadzot et al., 1989), and the 5-HT2a subtype appears to be of particular relevance as a selective 5-HT2a antagonist ketanserin effectively blocks most but not all of the characteristic effects of classical psychedelics (Kometer et al., 2013; Holze et al., 2021). This receptor type is densely distributed in the deep layers of the neocortex and believed to play major roles in coordinating high-order cognitive processing. According to the current knowledge serotonin receptors signaling plays roles in regulating aggression, anxiety, cognition, learning, memory, mood, sex, sleep, and thermoregulation. Moreover, effects on 5-HT receptor family are important parts for psychoactive properties of a wide variety of pharmaceutical substances, including antipsychotics, antidepressants, antiemetics, antimigraine agents, and anorectics (Nichols and Nichols, 2008).

However, the current treatment approaches for psychiatric conditions have limitations (Chisholm et al., 2016; Dell’osso and Lader, 2013). For example, antidepressants represent the first line pharmacological treatment for patients with major depressive disorder (MDD), a highly prevalent, disabling, antidepressants. However, 20% of patients with depression meet criteria for being considered treatment-resistant, with prolonged courses of illness and high relapse rates. (Gaynes, 2009). In addition to the issue of limited efficacy, there are other limitations to antidepressant use, such as intolerance to side effects and delayed therapeutic onset. (Penn and Tracy, 2012). In the attempt to provide sufficient care for all patients with mental health disorders, the amount of research on serotonergic psychedelics has increased significantly over the past decade (Carhart-Harris and Goodwin, 2017).

This resurgence of interest in the use of psychedelics in treating psychiatric disorders has been called a “renaissance” (Sessa, 2012). Psychoactive plants that cause alterations in consciousness, perception, thinking, and emotions have been used recreationally in various cultures for over 5,000 years (El-Seedi et al., 2005). It is suggested that naturally-occurring psychedelics have therapeutic potential due to the formation of new neural connections and modulation of the monoamine neurotransmitter system (Carhart-Harris et al., 2018; Erritzoe et al., 2018). Recent evidence suggests that psychedelics can benefit various clinical populations, with rapid and sustained advances in post-treatment outcomes documented for depression (Griffths and Grob, 2010; Begola and Schillerstrom, 2019), anxiety (Griffths and Grob, 2010), and addictions (Bogenschutz et al., 2015; Bogenschutz and Johnson, 2016). Importantly, the current evidence verified in animal studies has shown that classical psychedelics are not associated with physical dependence, abuse or withdrawal and have a low level of toxicity (Reiff et al., 2020).

In this review, we will discuss the three classic serotonergic naturally occurring psychedelics, psilocybin, ibogaine, and N, N-dimethyltryptamine (DMT), focusing on their pharmacological properties and clinical benefits. The purpose of this article is to provide a focused review of the research most relevant to the therapeutic potential to integrate the use of these psychedelics as a possible alternative or adjuvant to existing pharmacological and psychological therapies.

## Psilocybin

### Background

Psilocybin (4-phosphoryloxy-N,N-dimethyltryptamine) is a naturally-occurring hallucinogenic tryptamine alkaloid that was first isolated from Central American mushrooms (Psilocybe Mexicana) by the Swiss chemist Albert Hofmann in 1957 (Hofmann et al., 1959). A year later, psilocybin was chemically synthesized. Since then, numerous mushroom species (also known as “magic mushrooms”) genus Psilocybe, Panaeolus, Conocybe, Gymnopilus, Stropharia, Pluteus, and Panaeolina, have been found to contain psilocybin (Stamets, 1996; Cody, 2008; Tylš et al., 2014). For centuries psilocybin has been used by indigenous cultures as a means of spiritual exploration (Schultes and Hofmann, 1980) and has attracted particular attention in modern research for its psychoactive properties.

### Pharmacokinetics

“Magic mushrooms” are usually taken per os in the form of herbal preparations or smoked. The effective oral dose of psilocybin is about 0.045 mg/kg (Hasler et al., 1997). Depending on the individual’s body weight and the species of psychedelic mushroom, this can be an equivalent of 0.25–1.5 g of dry mushroom (Hasler et al., 1997).

After oral intake psilocybin is rapidly dephosphorylated to psilocin (4-hydroxy-N,N-dimethyltryptamine) in the acidic milieu of the stomach or the intestinal mucosa by both alkaline phosphatase and non-specific esterase (Tylš et al., 2014). Rapid dephosphorylation of psilocybin was determined after its parenteral administration (Johnson et al., 2008). Psilocin is thought to be the active agent in the CNS responsible for the manifestation of psychoactive effects, as it easily crosses the blood-brain barrier (Hasler et al., 1997; Johnson and Griffiths, 2017). Interestingly, both inactive psilocybin (dephosphorylated) and bioactive psilocin were naturally found in varying amounts in psychedelic mushrooms (Kamata et al., 2010). Since psilocin is structurally related to the neurotransmitter serotonin, it undergoes a comparable metabolism in the human body (Helsley et al., 1998). However, the complete metabolic pathway of psilocybin remains largely unknown, and much information must be obtained to identify the exact mechanisms implicated in its metabolism. After dephosphorylating, psilocin is subjected to either glucuronidation or oxidation and deamination (Passie et al., 2002). Glucuronidation occurs through the activity of 19 endoplasmic enzymes classified as uridine 5′-diphospho-glucuronosyltransferase UDP-glucuronosyltransferases (UGTs) (Manevski et al., 2010). In particular, psilocin glucuronidation is carried out by two UGT1A10 in the small intestine and UGT1A9 in the liver, resulting in the formation of the psilocin-O-glucuronide (glucuronide) conjugate (Manevski et al., 2010). The majority of the absorbed psilocin (approximately 80%) is eliminated from the body with urine (Kamata et al., 2003). Several pharmacologic studies have shown instant hydrolysis of psilocybin after a single oral dose to its active metabolite psilocin, which has a half-life of 163 min, and the presence of approximately 67% of psilocin glucuronide form in the urine (Hasler et al., 1997, Hasler et al., 2002). Thus, psilocin-O-glucuronide is the key urinary metabolite with clinical and diagnostic relevance. The remaining 20% of the absorbed psilocin is metabolized by oxidation, where the compound usually undertakes demethylation and deamination to form 4 hydroxyindole—3—acetaldehyde and 4—hydroxyindole—3—acetic acid or 4—hydroxytryptophol (Brown et al., 2017). These metabolites are excreted by the liver and kidney, however, enzymes involved in these reactions are unknown (Brown et al., 2017; Dinis-Oliveira, 2017). The half-life of psilocin is 3 h (±1.1 h) in healthy individuals, depending on specific characteristics and route of administration (Brown et al., 2017).

### Chemical Structure

**FIGURE 1:**
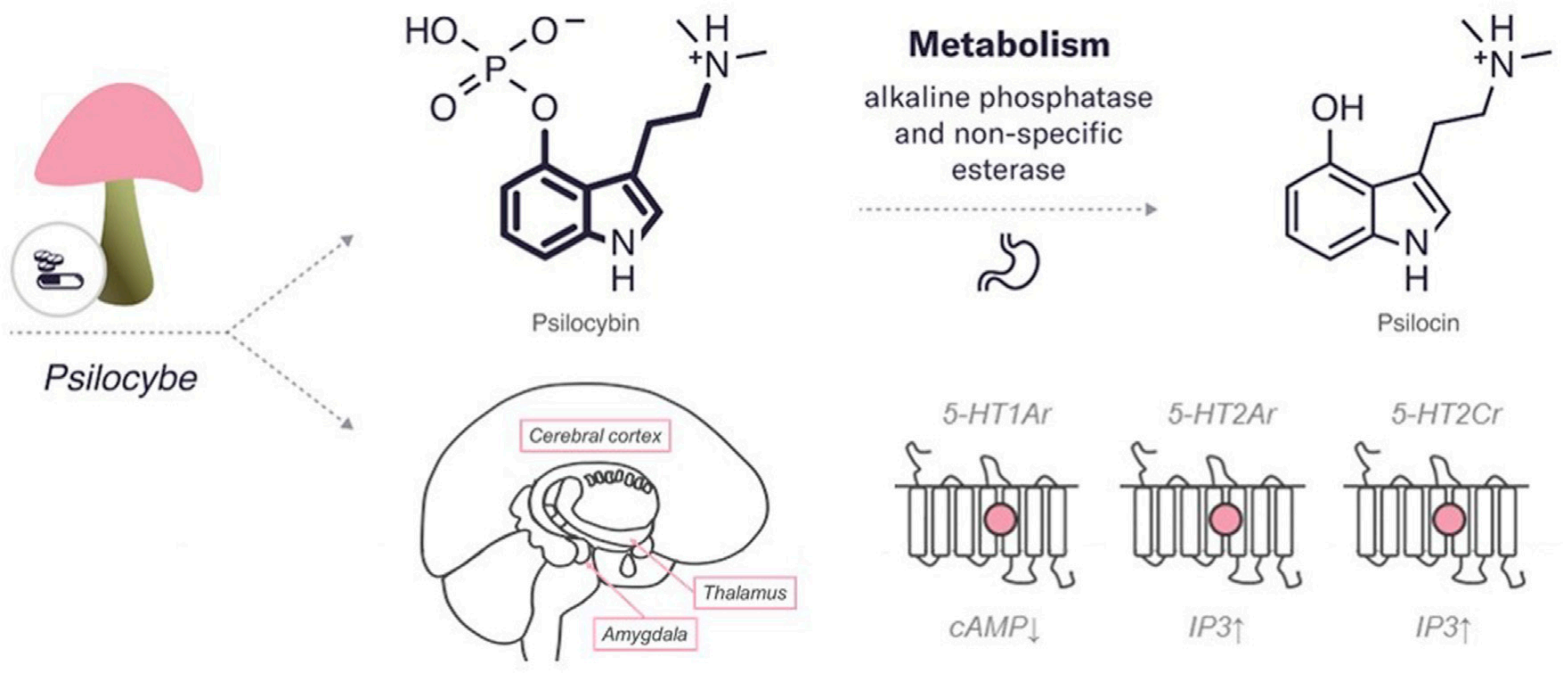
An overview of the characteristics of psilocybin. The diagram includes the chemical structures of psilocybin and its metabolite. The bold lines indicate similarities in the chemical structure between this psychedelic and serotonin. In addition, the enzymes and major organs involved in the metabolism and excretion of psilocybin are indicated. The mechanism of action represents the targets of the psychedelic, both the region of the brain affected by the psychedelic and the receptors with which it interacts (pink circle for agonist). 5HT—serotonin receptors (2Ar, 1Ar, 2Cr, – subtypes). IP3, inositol-3-phosphate; cAMP, cyclic adenosine monophosphate.

Psilocybin and psilocin, similarly to serotonin, have an indole ring structure, a fused double ring encompassing both a pyrrole ring and a benzene ring, linked to an amino group by a two-carbon side chain (Hasler et al., 2004) (refer to Figure 1). They differ from each other in position 4 carbohydrate, which contains phosphate and hydroxyl group, respectively.

### Mechanism of Action

Psilocybin is predominantly accountable for the psychedelic effects by a partial agonist action at serotonin receptors type 2A (5-HT2Ar) (González-Maeso et al., 2007; Nichols, 2016; Madsen et al., 2019). Pharmacological studies have demonstrated high-affinity binding of psilocin to 5-HT2Ar, with lower binding affinity to 5-HT1Ar and some other 5-HT receptor subtypes, such as 5-HT2Cr (McKenna et al., 1990; Rickli et al., 2016). In addition to mediating subjective and behavioral effects, these receptors are also involved in therapeutic effects. For example, altered cortical expression of 5-HT1Ar, 5-HT2Ar, and 5-HT2Cr was found in post-mortem samples obtained from depressed patients, suggesting that these receptors are associated with the dysfunctional shifts in emotional processing observed in these patients (Vollenweider and Kometer, 2010; Baumeister et al., 2014). Moreover, animal models and clinical studies have shown that 5-HT1Ar agonists have anxiolytic and antidepressant properties (Nutt, 2005; Katzman, 2009; Baumeister et al., 2014), and 5-HT2Ar and 5-HT2Cr agonists can reduce anxiety- and depression behaviour in animals (Nic Dhonnchadha et al., 2003; Baumeister et al., 2014). In particular, by activating 5-HT2Ar, psilocybin elevated positive mood, diminished recognition of negative facial expression, increased goal-directed behaviour toward positive versus negative cues, and supported positive emotional effects (Kometer et al., 2012; Mertens et al., 2020). The 5-HT2Ar agonism of psilocybin may be especially significant in the cerebral cortex due to the high expression of these receptors in the cortex. An increased expression of 5-HT2Ar was found in the post-mortem prefrontal cortex of depressed and suicidal patients (Pandey et al., 2002; Shelton et al., 2009). Several lines of evidence indicated that hallucinogenic 5-HT2Ar agonists, including psilocybin, induce the activity of intracellular signalling cascades in cortical pyramidal neurons, consequently modulating downstream signalling proteins, such as early growth response protein 1 and 2, and β-arrestin (González-Maeso et al., 2007). More recent studies have also shown the likelihood of high affinity for psilocybin to 5-HT2Ar in the cortex and the thalamus, mediating emotional processing of visual information (Muthukumaraswamy et al., 2013; Baumeister et al., 2014). Psilocybin administration resulted in desynchronization of ongoing oscillatory rhythms in the cortex, likely generated by 5-HT2Ar-mediated excitation of deep pyramidal cells (Muthukumaraswamy et al., 2013). Stimulation of 5-HT2Ar in the reticular nucleus may inhibit the thalamic filtering of information through GABAergic projections, possibly permitting cortical areas to receive more sensory information passing through (Baumeister et al., 2014). Moreover, psilocybin can reduce the metabolic activity of parts of the thalamus in humans (Gouzoulis-Mayfrank et al., 1999; Carhart-Harris et al., 2012), hence, causing sensory alterations associated with hallucinogens. These results suggest that accelerated processing of sensory information, as a consequence of reduced thalamic gating, may underlie psychedelic experiences induced by psilocybin. It can be suggested that altered integration of sensory perceptions allows for a unique experience of self and environment perception and may help reduce rigid thinking patterns as seen in psychiatric disorders. However, this hypothesis requires further verification in clinical populations. An additional study showed that psilocybin lowers connectivity between the right claustrum and brain structures that support perception, memory, and attention, such as the auditory and the default mode networks while increasing connectivity to the frontoparietal control network (Barrett et al., 2020). These data indicate that the subjective effects of psilocybin are linked to variations in both the amplitude of low-frequency fluctuations and the BOLD signal in the claustrum. Notably, the claustrum is a subcortical nucleus, in which 5-HT2Ar is highly expressed, and it provides glutamatergic inputs to possibly all cortical regions. Hallucinogen binding, psilocybin binding including, to postsynaptic 5-HT2Ar in cortical neurons, especially in cortical layer V, can cause changes in prefrontal glutamatergic signalling and cellular function through altered protein expression, with various manifestations in consciousness (Baumeister et al., 2014).

It has been proposed that metabotropic glutamate receptor 2/3 (mGluR2/3) ligands may be effective in modulating responses induced by 5-HT2Ar activation (Moreno et al., 2011). The formation of the 5-HT2Ar-mGluR2 complex implies a functional interaction that affects hallucinogen-regulated cell signalling pathways essential for the neuropsychological responses induced by hallucinogens, including psilocybin. Thus, the activation of mGlu2 secondary to 5-HT2A receptor-associated glutamate release emerged as the dominant mechanism of psilocybin action. Remarkably, the 5-HT2Ar and mGluR2/3 receptors display an overlapping distribution in the brain cortex (Marek et al., 2000). Indirect activation of glutamate networks by psilocybin increases neuroplasticity, particularly through the α-amino-3-hydroxyl-5-methyl4-isoxazole-propionic acid (AMPA) receptor, with a subsequent increase in cellular expression of downstream signalling protein, such as brain-derived neurotrophic factor (BDNF) in prefrontal areas (Vollenweider and Kometer, 2010). There is evidence for increased glutamate-dependent activity in prefrontal areas, induced by 5-HT2Ar agonism by psilocin and other classical hallucinogens (Béïque et al., 2007). Increased neuroplasticity of BDNF is implicated in neurotrophic theories of depression and its treatment (Caddy et al., 2014) since there is robust evidence that serum BDNF concentrations are decreased in depressed individuals and that antidepressant treatment regulates its levels (Duman, 2004; Sen et al., 2008). Besides, BDNF plays an important role in adult neurogenesis (Bath et al., 2012), and there is evidence that depression is related in part to insufficient neurogenesis and neurotrophic activity (Krishnan and Nestler, 2008). An animal study provided evidence that psilocybin helps to cope with fear far better than a placebo (USF Health News, 2013). This study supported the hypothesis that psilocybin can help break the trauma cycle that occurs in patients with post-traumatic stress disorder (PTSD). It has also been shown that psilocin can decrease amygdala reactivity in emotion processing through activation of postsynaptic 5-HT1Ar or 5-HT2Ar (Kraehenmann et al., 2015). Since patients with PTSD frequently exhibit increased amygdala reactivity (Francati et al., 2007), this may enhance the ability to process traumatic memories, highlighting the potential usefulness of psilocybin for PTSD therapy. The amygdala, which is intensely innervated by serotonergic neurons (Asan et al., 2013), is implicated in the processing of emotions and related memories. Furthermore, individuals with substances use disorder (SUD) have continual dysregulations in the emotional (amygdala-mediated) and stress (hypothalamic-pituitary-adrenal-axis-mediated) systems (Rao, 2010). These effects may be mediated through the serotonergic effects of psilocybin by binding to central 5-HT2Ar and 5-HT1Ar (de Veen et al., 2017). Moreover, the data indicating that BDNF in the mesocorticolimbic dopamine system *via* BDNF-Tropomyosin receptor kinase B transmission is a positive modulator of drug reward (cocaine, morphine, and amphetamine) and a negative modulator of alcohol reward were extensively reviewed (Ghitza et al., 2010). By modulating glutamatergic neurotransmission, psilocybin can affect the brain’s reward system, increasing cognitive and emotional control, which are the basis for reducing addictive behaviours (Catlow et al., 2013; Michael, 2013; Dos Santos et al., 2016).

In addition, the activity of psilocybin/psilocin has been described at a wide range of other receptors, including histamine-1, alpha-2A and -2B, and dopamine-3 receptors (Ray, 2010). Thus, the psychoactive effects responsible for the unique psychedelic profile observed in psilocybin users are achieved not only primarily due to partial agonism on cortical 5-HT2Ar and to a lesser extent on 5-HT1Ar and 5-HT2Cr, but also through activation of other receptors and receptor subtypes (De Gregorio et al., 2021). The pharmacological activity of psilocybin beyond 5-HT2Ar, 5-HT1Ar, and 5-HT2Cr has not been discussed here in detail. Overall, the therapeutic effects of psilocybin include serotonergic, glutamatergic, and dopaminergic signaling and local brain metabolic activity and functional connectivity between parts of the brain, such as the anterior and posterior cingulate cortex, thalamus, and amygdala.

Considering antidepressant effects, psilocybin exhibits some similarities to conventional antidepressants; on the other hand, they also retain some significant differences. In terms of similarities, altered relationships with the environment may be crucial to the recovery with selective serotonin reuptake inhibitors (SSRIs) (Harmer and Cowen, 2013; Belsky, 2016), and enhanced sensitivity to the environment is a key trait of the psychedelic state (Hartogsohn, 2016). In terms of differences, the long-term antidepressant action of SSRIs includes reduced limbic reactivity and emotional restraint or decline, likely achieved through post-synaptic 5-HT1Ar signalling (Cowen and Browning, 2015). This discriminates the superior role of 5-HT2Ar signalling in psilocybin use and emphasizes emotional release (Watts et al., 2017; Mertens et al., 2020). Different approaches to emotion may be the ultimate difference between the SSRI and psilocybin treatment models of depression and PTSD. However, there is a major challenge associated with taking SSRIs for long periods. The antidepressant effects of SSRIs and other conventional tricyclic antidepressants need at least 3–6 weeks to reach clinical efficacy (Frazer and Benmansour, 2002), and delayed pharmacological effects are undesirable in patients with terminal cancer. In contrast, a convincing characteristic of psilocybin therapy is the continued effect of a single high dose for the same period (Carhart-Harris et al., 2016; Carhart-Harris et al., 2018). Comparing the mechanisms and efficacy of psilocybin with conventional treatments is the next step in expanding the evidence base for psilocybin in depression.

### Safety

Although psilocybin toxicity is generally low, (Carbonaro et al., 2016), several adverse consequences related to psilocybin exposure were reported (Hasler et al., 2004). Various toxic effects were extensively reviewed (van Amsterdam et al., 2011; Nichols, 2016; Poulie et al., 2020), (refer to Table 1).

**TABLE 1:** The common adverse effects related to psilocybin exposure.

System	Side effects
Neurological and cognitive	Headache, confusion, euphoria, muscle weakness, hallucinations, panic attacks, loss of sense of reality, ego loss, illusions, synaesthesia, convulsions, changes in sense of thought and time, vertigo, anxiety
Respiratory	Transient hypoxemia
Gastrointestinal	Nausea, vomiting, diarrhoea
Renal	Acute kidney failure
Ocular	Mydriasis
Cardiovascular	Tachycardia, increased systolic blood pressure
Auditory	Sound enhancement and distortion
Other physiologic effects	Mild sedation with compulsive yawning, general stimulation, rhinorrhoea, hypersalivation, a slight rise in body temperature

Notably, a survey showed that these adverse effects were often seen in people who used the mushrooms, but not in those who consumed isolated psilocybin (Carbonaro et al., 2016). More severe adverse events include hallucinogen persisting perception disorder (Orsolini et al., 2017), seizures (Jo et al., 2014), and a hypothetical risk of the development of stiffening of cardiac valves with frequent long-standing use (Löfdahl et al., 2016). Related to the latter, there has been one anecdotal case of a psilocybin-related death of a heart transplant patient (Lim et al., 2012). In addition, some fatal cases have been reported as a result of severe emotional destabilization or hallucinations leading to suicidal behaviour, such as the conviction of the ability to fly (Müller et al., 2013). However, in 2015, a study has found that those who used a classic psychedelic [i.e., psilocybin, N,N-dimethyltryptamine, lysergic acid diethylamide (LSD), and mescaline] in the past year had a reduced risk of attempted suicide by 36% (Hendricks et al., 2015). Also, in contrast to popular belief that using classic psychedelics, including psilocybin, can increase the risk of psychiatric illnesses, such as schizophrenia (Quednow et al., 2010), psilocybin is found to be unrelated to mental health problems (Johansen and Krebs, 2015).

Based on the known pharmacodynamics properties of psilocybin, its specific interactions with some drugs have been observed in some studies and described by multiple anecdotal reports. Some substances, such as monoamine oxidase inhibitors, can intensify the hallucinogenic effects of psilocybin, so psilocin abusers take them simultaneously (Halpern, 2004). There is another substance, such as caffeine, which is not a synergist of psilocybin; nonetheless, it may increase undertones of stimulation, changing the course of the experience as a whole (Temple et al., 2017). Moreover, the most dangerous known psilocybin interaction is with tramadol, which can reduce the seizure threshold (Fujimoto et al., 2015). Several wide-use drugs, including ethanol, gammahydroxybutyric acid, SSRIs, and benzodiazepines, may weaken the effects of psilocybin (Carey and Babu, 2015). According to Erowid Experience Vaults: Mushroom Reports, two drug-to-drug interactions that can generate distinctive effects, when co-administered with psilocybin, are cannabis and amphetamines. Thus, when used under the influence of psilocybin, cannabis can cause either relaxation or severe anxiety. Amphetamines can increase the risk of a “thought loop” (i.e., when the user is trapped in a certain sequence of thoughts or ideas). These effects appear to vary not only between individuals but also between experiences. It is important to remember that, despite the limited data available, there is always the possibility of an interaction of psilocybin with drugs and food.

Nonetheless, psilocybin appears well tolerated by healthy individuals and clinical populations, and rapid tolerance to continual use may decrease the risk of addiction (van Amsterdam et al., 2011; Baumeister et al., 2014). Moreover, a systematic review summarized evidence obtained from clinical research, observational studies, drug harm or risk assessments, and pharmacological studies that suggests low toxicity and reasonable safety of psilocybin when administered in supervised or controlled settings (Dos Santos et al., 2016). Researchers at Johns Hopkins University recently assessed the abuse potential of medically administered psilocybin (Johnson et al., 2018). They determined that if psilocybin were approved as a medication, it could be classified as a Schedule IV drug.

### Therapeutic Effects in Mental Health Disorders

The effects of psilocybin and its active metabolite psilocin on human behaviour are well studied and include various physiologic, visual, auditory, cognitive, emotional, transpersonal, perceptual, and multisensory alterations (Griffiths et al., 2011; Tylš et al., 2014; Kargbo, 2020). It is worth mentioning that the general effects of psilocybin use are usually dose-dependent, and there is a significant difference between the effects of high and low doses of psilocybin. For example, a double-blind, placebo-controlled dose-effect study on eight healthy volunteers demonstrated that high doses of psilocybin caused intense excitement with significant visual distortion. In contrast, low doses had a mild sedative effect with greater visual sharpness (Hasler et al., 2004). Besides, in healthy subjects, psilocybin was shown to generate positive subjective alterations in mood, perception, and psychological states in a dose-dependent manner (Studerus et al., 2011). In this study, acute adverse drug reactions, including strong dysphoria and/or anxiety/panic attack, appeared following the two highest dose conditions in a rather small proportion of subjects. Although the psilocybin dose is the major determinant of the acute psychedelic experience, numerous studies have shown a high degree of inter- and intra-individual variability in subjective responses to psilocybin that is currently not well understood (Haijen et al., 2018; Russ et al., 2019). Moreover, the quality of the psilocybin-generated experience is not only governed by dose, but also can be influenced by non-pharmacological factors, such as individual`s psychological, social, and cultural parameters, as well as the venue of psilocybin administration (Studerus et al., 2011; Hartogsohn, 2017).

The potential beneficial and therapeutic effects of psilocybin in mental health remain completely unexplored, therefore, its controlled medical use remains controversial. Nevertheless, in the past two decades, psilocybin has been increasingly investigated as a promising treatment for many different mental illnesses (Carhart-Harris and Goodwin, 2017). It is still unclear to what extent neurobiological mechanisms or the psychological experience of an altered state of consciousness, involving personally meaningful and spiritually significant mystical-type experiences, are responsible for the positive therapeutic effects. Quality experiences are associated with positive changes in mood, attitude, and behaviour in healthy individuals (Studerus et al., 2011; Griffiths et al., 2018; Madsen et al., 2020), as well as with positive therapeutic outcomes in patients with alcohol dependence (Bogenschutz et al., 2015), tobacco addiction (Garcia-Romeu et al., 2015), obsessive-compulsive disorder (Moreno et al., 2006), treatment-resistant depression (TRD) (Roseman et al., 2018), and cancer-related psychiatric disorder (Agin-Liebes et al., 2020).

Three double-blind randomized controlled trials examined the impact of a single dose of psilocybin on symptoms of anxiety and depression, which are common in cancer patients (Grob et al., 2011; Griffiths et al., 2016; Ross et al., 2016), and an open-label trial of psilocybin for TRD was accomplished (Carhart-Harris et al., 2016). In all these studies, rapid, noticeable, and persisting anti-anxiety and anti-depression effects were observed after psilocybin use, demonstrating promise for the treatment of these conditions. Besides, the recent findings obtained by (Carhart-Harris et al., 2018) corroborate the results of the mentioned-above TRD study supporting the efficacy and safety of psilocybin for treating symptoms of MDD. In this open-label clinical trial evaluating psilocybin with psychological support in the TRD study, twenty patients with severe unipolar TRD were administered two oral doses of psilocybin (10 and 25 mg, with 7-day intervals) under a supportive setting. Significant reductions in depressive symptoms were registered during the first 5 weeks after treatment. Thus, the accumulated evidence to date indicates that psilocybin may provide a single-dose treatment model with rapid and robust effects in depressive illness. The US Food and Drug Administration (FDA) awarded “breakthrough therapy” status to a psilocybin treatment developed by London-based Compass Pathways Ltd. for TRD, advancing the development process in 2018 (Compassion, 2021). Subsequently, the FDA also designated “breakthrough therapy” status to psilocybin treatment for MDD in trials financially supported by the Usona Institute (Usona Institute, 2021). Furthermore, the antidepressant, anxiolytic, and positive effects in emotional processing of psilocybin strongly support its potential therapeutic role in the treatment of PTSD (Kraehenmann et al., 2015; Pokorny et al., 2017; Roseman et al., 2019; NYU Langone Health, 2021). Also, psilocybin was demonstrated to increase some personality traits, such as insightfulness (Kometer et al., 2015) and openness (MacLean et al., 2011) that may be relevant to PTSD treatment. However, the beneficial effects of psilocybin in PTSD have to be verified in clinical trials, which are currently lacking.

In addition, psilocybin therapeutic use can provide an exceptional advantage as a possible monotherapy in SUD. For example, mystical-type experiences of psilocybin were demonstrated to mediate its therapeutic effects in nicotine addiction. In a recent open-label pilot study of psilocybin-facilitated smoking dependence therapy, in which 15 smokers received 2 or 3 doses of psilocybin in the setting of cognitive behavioural therapy for smoking cessation (Garcia-Romeu et al., 2015). The majority (12 of 15 participants or 80%) showed biologically verified smoking abstinence at a 6-month follow-up. Also, a single-group proof-of-concept study was conducted to quantify acute effects of psilocybin in alcohol-addicted participants, with positive outcome and safety data (Bogenschutz et al., 2015). Ten volunteers with severe alcohol dependence received orally administered psilocybin in one or two supervised sessions and abstinence increased significantly post-administration (*p* < 0.05). In these participants, psilocybin was applied in addition to psychotherapy.

## Ibogaine

### Background

Ibogaine (10-methoxyibogamine)—an indole alkaloid—found in *Tabernanthe iboga*, *Voacanga africana*, and *Tabernaemontana* undulata plants belonging to the Apocynaceae family (Koenig and Hilber, 2015). Ibogaine is the most abundant alkaloid in Tabernanthe iboga; 5%–6% in the fresh plant and 1%–2.6% in the dried root relative to the content of other indole alkaloids. The property of ibogaine to oxidase in solution may explain the higher value of the alkaloid in the fresh plant (Pope, 1969). The chemical synthesis of ibogaine was first described in 1957 by Morrice-Marie Janot and Robert Goutarel in a US patent titled “Derivatives of the ibogaine alkaloids” (Derivatives of the ibogaine alkaloids, 1957). Ibogaine has been studied according to its three main properties: 1) a cholinesterase inhibitor, 2) a deep stimulant, and 3) a hallucinogen (Alper et al., 2001). The hallucinogenic and psychoactive properties of ibogaine have been used for centuries in religious rituals, spiritual rides, and medicine, but were underestimated until the 1970s. To date, ibogaine is considered one of the most promising drugs for treating SUD (Belgers et al., 2016) by reducing substance dependence (alcohol, opioid or psychostimulant) withdrawal symptoms, and drug-seeking behaviour (Staley et al., 1996; Cameron et al., 2021).

### Chemical Structure

**FIGURE 2:**
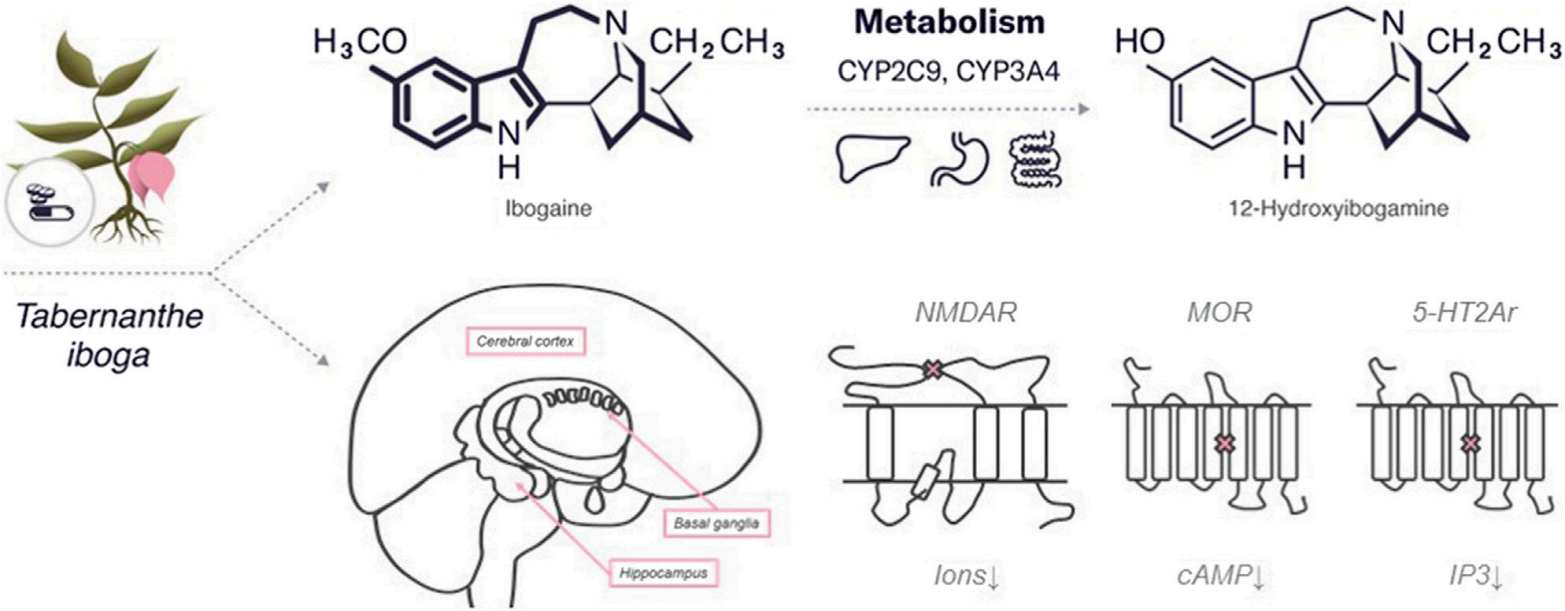
An overview of the characteristics of ibogaine.

Ibogaine is a psychedelic substance that belongs to the class of tryptamines along with both psilocybin and psilocin. Similar to psilocybin, it has an indole ring containing two fused rings, benzene and a pyrrole but with an additional cyclic structure. The molecular formula of ibogaine is C20H26N2O with a molar mass of 310.441 g/mol. Ibogaine has two chiral centers, which determine its four different isoforms. The main metabolite of ibogaine is noribogaine, which has the molecular formula C19H24N2O and a molar mass of 296.414 g/mol. Noribogaine has the same chemical structure as ibogaine but without one methyl group (refer to Figure 2).

### Pharmacokinetics

Ibogaine can be administered by different routes, as an oral suspension and solution, an intravenous injection (i.v.), and an intraperitoneal injection (i.p.). The absorption of ibogaine has been studied in human and animal models using rats, beagle dogs, and cynomolgus monkeys of both sexes (Alper, 2001). Ibogaine bioavailability data indicated that its absorption is nonlinear and has gender-related differences (Alper, 2001).

Some studies using rodent models demonstrated the ability of ibogaine and its metabolite to accumulate in organs and tissues. After an i.v. of 10 mg/kg, ibogaine mainly enters the blood plasma and reaches its maximum concentration in the brain (∼133 μM) in 10 s (Zetler et al., 1972). A higher i.p. dose of 40 mg/kg of ibogaine resulted in the distribution in the brain, kidney, liver, and plasma (Kubilienė et al., 2012). As a lipophilic substance, ibogaine can accumulate in adipose and heart tissues. An intravenous administration resulted in a 100-fold increase in the concentration of ibogaine in adipose tissue and a 30-fold—in the heart (Hough et al., 1996). Ibogaine persistence in fat may contribute to its long-term action.

The greater part of ibogaine biotransformation occurs in the liver *via* cytochrome CYP2D6—a member of the CP450 cytochrome family—provided O-demethylation of ibogaine to its major metabolite noribogaine (Obach et al., 1998; Litjens and Brunt, 2016). Other members of CP450, such as CYP2C9 and CYP3A4, are also involved in the conversion of ibogaine to noribogaine (Litjens and Brunt, 2016). Besides ibogaine metabolism in the liver, the intestinal wall also can be involved in this process (Obach et al., 1998). The kidneys and gastrointestinal tract excrete approximately 90% of both ibogaine and noribogaine (Mash et al., 2000). The half-life of ibogaine is 1–2 h in rodents (Zubaran et al., 1999) and in humans, 7.45 h (Glue et al., 2015) with following complete extraction during 24 h (Mash et al., 1998).

### Mechanism of Action

The antiaddictive properties of ibogaine are related to the neurotransmitter system and are mediated by various mechanisms of action. The specific target or affected pathway of ibogaine remains unknown, but the affinity of ibogaine for various CNS receptors, including N-methyl-D-aspartate (NMDA) receptors (Chen et al., 1996), κappa and µ-opioid receptors (MOR), (Codd, 1995; Antonio et al., 2013), and sigma-2 receptor sites (Bowen, 2000) has been established in several studies. As a non-competitive antagonist, ibogaine may interact with nicotinic acetylcholine receptors (nAChR) (Mah et al., 1998) providing their prolonged depolarization (Arias et al., 2010). Noribogaine is a weak NMDA receptor antagonist and also can bind opioid receptors with higher affinity than ibogaine (Barceloux, 2012). Both ibogaine and noribogaine can have a summating effect on the CNS (Belgers et al., 2016).

Ibogaine, a competitive antagonist, can induce slow, concentration-dependent blockage of NMDA receptor-coupled ion channels (Ki: ∼1 μM) (Chen et al., 1996). In contrast, ibogaine does not affect AMPA, kainate and glutamate receptors, and the metabotropic sites of NMDA receptors. NMDA receptors are essential for the normal brain function mediating excitatory signalling. Dysfunction of NMDA receptors is related to schizophrenia, autism spectrum disorders, mood disorders, including depression, bipolar disorder, SUD, and psychosis (Yamamoto et al., 2015). It has been shown that ibogaine can bind within the NMDA channel, similarly to other NMDA receptor antagonist dizocilpine, and prevent the ion flow through the channel (Chen et al., 1996). Alteration in ion equilibrium is linked to the development of psychiatric disorders and the antidepressant effect of ibogaine (Rodrı Guez et al., 2020). The interaction of ibogaine with NMDA receptors may explain its specific properties, such as antiaddictive effect, i.e., blocking the rewarding and reinforcing effects of drugs, such as morphine and cocaine (Schenk et al., 1993; Tzschentke and Schmidt, 1995).

Ibogaine NMDA interaction may decrease dopamine levels and increase concentrations of dopamine metabolites, such as dihydroxyphenylacetic acid and homovanillic acid (Popik et al., 1995). Therefore, ibogaine may indirectly affect the dopaminergic system that plays key roles in reward and prediction-error signaling (Arias-Carrión et al., 2010).

Ibogaine interacts with the neurotransmitter system by structurally binding to serotonin receptors, serotonin (SERT) and dopamine transporters (DAT) (Chen et al., 2020). Ibogaine binds to 5-HT1Ar, 5-HT2Ar, and 5-HT3r with low affinity (Ki: > 100, 12.5, and >100 μM, respectively) (Repke et al., 1994), and half-maximum inhibitory concentration (IC50) values of about 4 μM for the radioligands 5-HT2Ar and 5-HT3r (Sweetnam et al., 1995). Animal studies have shown that ibogaine injections increase serotonin levels in the nucleus accumbens (Wei et al., 1998) and striatum (Ali et al., 1999) and decrease levels of the serotonin metabolite 5-hydroxy-indoleacetic acid (5-HIAA) in the frontal cortex, hippocampus, olfactory tubercle (Sershen et al., 1992), striatum (Benwell et al., 1996), and the nucleus accumbens (Ali et al., 1999). The opposite effect has been demonstrated in the medial prefrontal cortex, where administration of ibogaine (40 mg kg−1, i.p.) resulted in decreased 5-HT levels and increased 5-HIAA levels (Benwell et al., 1996). Serotonin receptors are G-protein-coupled receptors involved in different signal transduction pathways. Differences in their action may explain the following effects of ibogaine on the brain: 1) 5-HT1r is associated with Gi/Go-protein and decreased cellular levels of cyclic adenosine monophosphate (cAMP); 2) 5-HT2r with Gq/G11-protein and increased cellular levels of inositol trisphosphate and diacylglycerol; 3) 5-HT3r is a ligand-gated NA+ and K+ cation channel, the activation of which leads to polarization of the plasma membrane (Nichols, 2016). Besides, ibogaine affects 5-HT1r through significant inhibition of adenylyl cyclase activity in the hippocampus (Rabin and Winter, 1996).

Apart from the interaction with 5-HTr, ibogaine binds SERT and DAT with higher affinity (Ki: ∼10 and ∼2 μM, respectively) (Mash et al., 1998; Asjad et al., 2017), depending on transporter’s conformation—in the outward-facing conformation of the transporter ibogaine binding is 10 times weaker than the inward-facing conformation. Thus, ibogaine can bind to DAT in the inward-facing conformation and can stabilize its tertiary structure, including misfolded mutant variants (Coleman et al., 2019).

Ibogaine binds allosterically to nicotinic and muscarinic AChR—ligand-gated ion channels, as a non-competitive inhibitor (Deecher et al., 1992). Some studies have shown that ibogaine inhibits ligands binding to M1, M2, and M3 (Repke et al., 1994). Ibogaine interacts withα1β1 and α3β4 subtypes (Ki: ∼0.4–1 μM) expressed in the habenulo-interpeduncular cholinergic brain pathway, which is known as the second drug reward pathway (Arias et al., 2010). Ibogaine potently blocks sodium chloride influx through nicotinic receptor channels with long-lasting effect (Popik et al., 1995). By inhibition of nAChR, ibogaine may lead to decreased catecholamine release and further drug-seeking behaviour (Mah et al., 1998).

Both kappa and µ-opioid receptors (MOR), as well as nAChR, are closely associated with the development of addiction in SUD (Rahman et al., 2015; Belzeaux et al., 2018). Ibogaine binds to MOR with relatively high affinity (Ki ∼10–100 μM), suggesting that ibogaine is not an orthosteric MOR agonist (Glick et al., 1994), while binding to kappa-opioid receptors has a lower affinity (Ki: ∼2–4 μM) (Sweetnam et al., 1995). Besides, the highest affinity of ibogaine binding to the µ-opioid agonist site was found in the presence of KCl or N-methyl-D-glucamine hydrochloride (Codd, 1995).

Alike 5-HTr, MOR is a G-protein-coupled receptor that inhibits adenylate cyclase (AC) activity and reduces cAMP levels (De Vries et al., 1990). Ibogaine acts downstream of G-protein-coupled receptor activation, possibly directly on AC. Self-administration of ibogaine showed no effect of analgesia in preclinical models but may potentiate morphine analgesia (Francés et al., 1992; Bhargava et al., 1997). In contrast, noribogaine, the most potent serotonin reuptake inhibitor, can reduce tolerance to morphine analgesia in animal models (Sharma et al., 2007). These contrasting effects of ibogaine and noribogaine are specific only to MOR but not for kappa or delta opioid agonists (Cao and Bhargava, 1997). This interaction determines the effects of ibogaine treatment on opioid withdrawal, which cannot be explained by MOR-coupled G-protein activation (Rabin and Winter, 1996; Antonio et al., 2013). Taken together, interaction of aforementioned receptors with ibogaine can reduce drug cravings, decrease withdrawal symptoms, and prevent relapse.

In addition, acute ibogaine injections alter the expression of certain proteins involved in normal brain function, such as substance P (Alburges et al., 2009), BDNF, and glial cell-derived neurotrophic factor (GDNF), in a dose- and time-dependent manner, supporting ability of ibogaine to attenuate drug-seeking behaviour (Popik et al., 1995; Marton et al., 2019).

### Safety

Acute administration of a 25 mg/kg (the high dose) of ibogaine showed a neurotoxic effect on Purkinje cells in the rat cerebellum (O’Hearn and Molliver, 1993; Helsley et al., 1997), while no toxicity has been observed with long-term (during 60 days) i.p. administration of 10 mg/kg of ibogaine (Helsley et al., 1997). According to animal studies, administration of ibogaine can cause complex side effects: tremor associated with inferior-olive mediated neurotoxicity in the cerebellum (O’Hearn and Molliver, 1993; Miwa, 2007); gender-dependent locomotor activity (Sershen et al., 1992); slower response times on sensory and sensory-motor tests and deterioration of specific motor reflexes (Kesner et al., 1995); fear-like reactions (Schneider and Sigg, 1957); anxiety (Gershon and Lang, 1962); attenuated acquisition of spatial memory (Helsley et al., 1997); increased blood pressure and heart rate (HR) (Gershon and Lang, 1962).

In humans, the side effects of ibogaine on the cardiovascular system may be a crucial limitation for further investigation of its antiaddictive properties. Ibogaine can decrease HR and cause life-threatening cardiac arrhythmias (Koenig et al., 2014) that have to be controlled during treatment. The use of ibogaine has also led to several reported cases of sudden death with an unclear cause (Maas and Strubelt, 2006; Alper et al., 2012; Aćimović et al., 2021). This may be related to a blockage of human ERG potassium channels in the heart and further abrupt prolongation of QT interval on electrocardiogram associated with ventricular tachyarrhythmias (Koenig et al., 2014). In addition, therapeutic concentrations of ibogaine may lead to tachycardia (Koenig and Hilber, 2015; Litjens and Brunt, 2016). Thus, the implementation of ibogaine for therapeutic use requires further studies on its effects on the cardiovascular system.

### Therapeutic Effects in Substances Use Disorder

The first positive results on addiction treatment with ibogaine were obtained by Howard Lotsof in 1962 and 1963, when he conducted an experiment on a group of twenty Caucasian college men, seven of whom had a heroin addiction and opioid withdrawal symptoms (Alper et al., 2001). After ibogaine treatment, five of those seven maintained abstinence for 6 months or longer. The crucial limitation of this experiment was the self-reporting of a small group of participants. Since then, there has been a significant increase in the number of published and approved studies that have evaluated the positive antiaddictive effects of ibogaine administration. Thus, ibogaine has become a promising candidate for the treatment of addiction. In animal studies, a dose-dependent reduction in self-administration of cocaine (Glick et al., 1994), morphine (Glick et al., 1991), heroin (Dworkin et al., 1995), and withdrawal symptoms has been recorded after ibogaine use. Ibogaine also reduced alcohol intake in alcohol-preferring Fawn Hooded rats (Rezvani et al., 1995). Moreover, ibogaine administration reduced some signs of withdrawal (jumping, rearing, digging, head hiding, chewing, teeth chattering, writhing, and penile licking) in animal and human morphine dependence (Popik et al., 1995).

Recent studies showed that taking ibogaine led to a reduction in opiate craving and a reduction in the signs and symptoms of drug withdrawal (Davis et al., 2017; Mash et al., 2018). Furthermore, ibogaine has shown to be a promising treatment for opiate, heroin, cocaine, and alcohol dependence, reducing withdrawal symptoms and promoting continued abstinence (Brown, 2013). In particular, opioid use disorder (OUD) is based on opioid dependence, which poses an economic, social, and health care burden. Currently, there is an enormous crisis in North America related to the uncontrolled use of opioid drugs (Fischer et al., 2020). The classical treatment for opioid dependence is Methadone Maintenance Therapy (MMT), established on methadone to effectively reduce heroin use (Oviedo-Joekes et al., 2009; Mattick et al., 2014). However, many patients on MMT need alternative compounds, such as ibogaine, to treat OUD because of the limited efficacy of MMT. Several studies have reported that up to 40% of patients with OUD have an adverse response to MMT (Oviedo-Joekes et al., 2009; Mattick et al., 2014). The utility of ibogaine for treating OUD has been demonstrated. One study presented the case of a 37-years old Caucasian woman with severe OUD who achieved 18 months of sustained opioid cessation after 4 days of ibogaine treatment with some mild and transient side effects, such as weakness, dizziness, and diaphoresis (Cloutier-Gill et al., 2016). In another study, opioid-dependent patients treated with ibogaine showed significant reductions in scores on various mental health tests after a single dose of ibogaine (Noller et al., 2018). In this study, ibogaine contributed to a reduction in withdrawal symptoms and a sustained reduction or even cessation of opioid use, and these effects persisted for 12 months. Additionally, the antiaddictive effects of ibogaine were studied in 75 users of alcohol, cocaine, crack, and cannabis. Schenberg et al., 2014 showed that a single dose of ibogaine could lead to significant long-term abstinence from drugs without any fatalities (Schenberg et al., 2014).

Despite positive findings, ibogaine is still a Schedule I drug in the US, which remains unavailable for medical use due to its high potential for abuse.

## Dimethyltryptamine

### Background

Humans have consumed N,N-Dimethyltryptamine (DMT or N,N-DMT), a key ingredient of ayahuasca, in various tisanes and snuffs used during religious ceremonies in Central and South America for centuries (Barker, 2018). Moreover, the use of ayahuasca, a psychoactive plant brew, containing leaves of Psychotria viridis, Diplopterys cabrerana, Psychotria carthagenensis, and the root bark of Mimosa tenuiflora is associated with the traditional practices of the indigenous people of the North-Western Amazon region. Accounts of such rituals indicate that, after consuming these plant mixtures, people felt peaceful and enlightened, most likely due to the profound psychoactive effects of chemical components of the brew. Of the natural components in these mixtures, DMT has garnered significant interest because of its intense hallucinogenic effects in humans. DMT was also found in fungi, marine sponges, tunicates, frogs, legumes, and grasses (Hamill et al., 2019). It can be produced endogenously in human and rat brains (Dean et al., 2019) and it has been detected in human urine, blood, and cerebrospinal fluid (Carbonaro and Gatch, 2016). In the past several decades, there has been growing international interest in its possible therapeutic effects (Shanon, 2002; Labate, 2014).

### Chemical Structure

**FIGURE 3:**
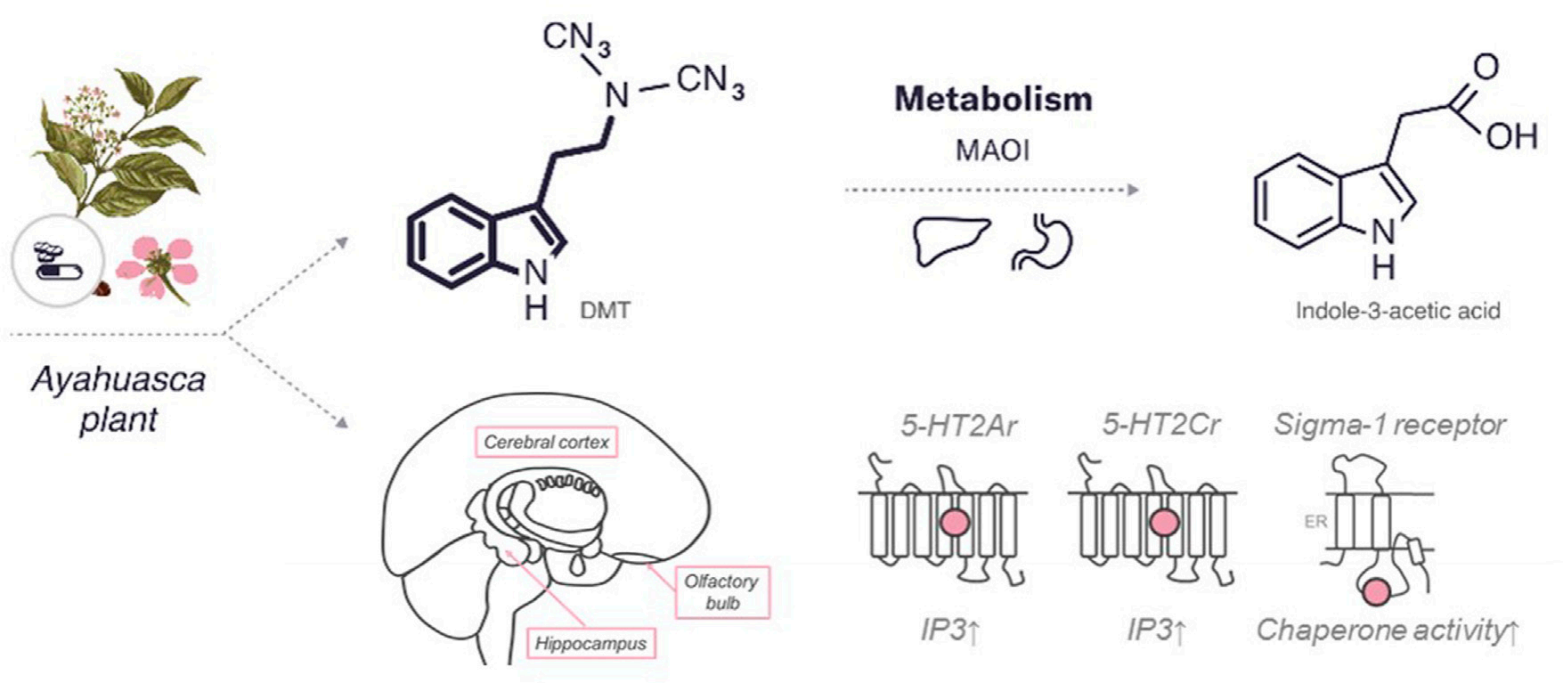
An overview of the characteristics of DMT. The diagram includes the chemical structures of DMT and its metabolite. The bold lines indicate similarities in the chemical structure between this psychedelic and serotonin. In addition, the enzymes and major organs involved in the metabolism and excretion of psilocybin are indicated. The mechanism of action represents the targets of the psychedelic, both the region of the brain affected by the psychedelic and the receptors with which it interacts (pink circle for agonist). 5HT—serotonin receptors (2Ar, 2Cr,—subtypes), sigma 1 receptor. MAOI, monoamine oxidase I; IP3, inositol-3-phosphate; ER, endoplasmic reticulum.

DMT is a derivative, functional and structural analogue of the following tryptamines: O-acetylpsilocin (4-AcO-DMT), 5-methoxy-N,N-dimethyltryptamine (5-MeO-DMT), psilocybin (4-PO-DMT), psilocin (4-HO-DMT), and bufotenin (5-HO-DMT) (Malaca et al., 2020). The chemical structure of DMT comparable to indole-containing serotonergic psychedelics, such as psilocybin and ibogaine, is presented in Figure 3.

### Pharmacokinetics

DMT has a rapid onset of action, intense effects, and a relatively short duration of action. DMT can be inhaled, ingested or injected and its effects depend on the dose and administration route.

The metabolism and pharmacokinetics of DMT play a prominent role in how it is typically administered and why it produces a qualitatively different experience than other psychedelics. Ayahuasca produces psychedelic effects lasting approximately 4 hours, with peak cognitive lasting 60–120 min (Hamill et al., 2019). Compared to ayahuasca, smoked, i.v., and insufflated DMT has a very rapid onset of action, with peak cognitive effects lasting 3–10 min and episodes lasting 5–15 min. The threshold dose for hallucinogenic effects of DMT is 0.2 mg/kg when administrated intravenously.

The maximum plasma concentration (Tmax) is about 1.5 h (Riba et al., 2003) for both high and low doses of DMT (Hamill et al., 2019). Only 1.8% and 0.16% of the injected dose of DMT can be measured in human blood and urine, respectively, at any given time (Strassman and Qualls, 1994; Barker, 2018). In rats, the accumulation of DMT occurs in the cortex and amygdala, brain structures that play a key role in behaviour (Mandell and Morgan, 1971; Dean et al., 2019). DMT has been shown to accumulate in brain slices through an active transport mechanism that can be saturated, sensitive to metabolic inhibitors, and temperature-, glucose-, and sodium-dependent (Sangiah et al., 1979; Cameron and Olson, 2018).

In addition to rapid access to brain tissue following the systemic administration, DMT is rapidly metabolized by monoamine oxidase A (MAO-A), as well as by liver enzymes (Simão et al., 2019). The *in vivo* half-life of DMT is approximately 5–15 min and can be extended by the treatment with a MAO inhibitor (Cameron and Olson, 2018). DMT is inactive after oral ingestion due to a rapid degradation under the influence of MAO-A in the intestine and liver (Barker, 2018). In the case of ayahuasca use, the tisanes can be ingested because it also contains MAO-A inhibitors like harmine, enabling sufficient amounts of orally-administered DMT to reach the brain.

### Mechanism of Action

Unmetabolized DMT interacts with various receptors in the brain, including a large number of serotonin receptors. DMT is known mostly for its activity as a 5-HT2Ar agonist (Aghajanian and Marek, 1999; Barker, 2018). It also has a nanomolar affinity for 5-HT1A/1B/1D/2A/2B/2C/6/7 receptors (Halberstadt and Geyer, 2011), with proven agonist or partial agonist activity at 5-HT1A/2A/2C receptors (Smith et al., 1998). Psychedelic effects are mediated mainly by 5-HT2A/2C receptors (Cumming et al., 2021).

The 5-HT2Ar—a Gq-coupled protein—is found in many mammalian brain regions: in the cortex, striatum, hippocampus, and amygdala, with a particularly high expression on layer V pyramidal neurons of the cortex (Zhang and Stackman, 2015). DMT acts as a 5-HT2Ar agonist, causing an increase in phosphoinositide hydrolysis, as well as in both the frequency and amplitude of spontaneous excitatory postsynaptic currents in layer V cortical pyramidal neurons (Ly et al., 2018). The small methyl groups of DMT are critical for achieving high affinity for 5-HT2Ar, and this receptor does not desensitize to DMT over time, that may explain why humans do not develop tolerance to DMT (Smith et al., 1998).

Stimulation of 5-HT2Ar may be the mechanism behind the psychoplastogenic effects of DMT. It increases the complexity of the dendritic arbours of cortical neurons and promotes an increase in dendritic spine density through an mTOR-dependent mechanism that involves 5-HT2Ar activation (Ly et al., 2018). Neural plasticity in the prefrontal cortex is crucial for behavioural effects of fast-acting antidepressants, for example, ketamine, so it is possible that 5-HT2Ar underlies the known antidepressant effects of serotonergic psychedelics, including DMT.

Like 5-HT2Ar, 5-HT2Cr binds to Gq and increases phosphoinositide hydrolysis upon activation. DMT acts as a partial agonist of 5-HT2Cr with a binding affinity approximately half that of 5-HT2Ar. However, unlike 5-HT2Ar, 5-HT2Cr desensitizes to DMT over time (Smith et al., 1998). In contrast to 5-HT2Ar and 5-HT2Cr, 5-HT1Ar are inhibitory G-protein-coupled receptors expressed on cells located mainly in cortical and subcortical regions (Leiser et al., 2015). These receptors can also serve as autoreceptors located on somas and dendrites of serotonergic neurons in the dorsal raphe (Garcia-Garcia et al., 2014). DMT is a good ligand for 5-HT1Ar (183 nM) compared with its affinity for other receptors (Halberstadt and Geyer, 2011) where it acts as an agonist. Increased activation of these autoreceptors can reduce serotonin release in other brain regions, but chronic treatment with antidepressants restores serotonin neuron activity by desensitization of somatodendritic and terminal autoreceptors (Le Poul et al., 1995; Cremers et al., 2000; Cornelisse et al., 2007). This is because many 5-HT1Ar agonists are thought to exert anxiolytic and antidepressant properties, and in the case of DMT, this mechanism may also contribute to its therapeutic effects.

Finally, there are reports that DMT also binds to 5-HT1D, 5-HT6, and 5-HT7 receptors with high affinity (39, 464, and 206 nM, respectively) (Cameron and Olson, 2018), but little work has been done to fully characterize the interaction of DMT with these receptors beyond initial binding studies. A wide variety of 5-HT1D, 5-HT6, and 5-HT7 ligands possess DMT-containing backbones (Cameron and Olson, 2018).

The sigma-1 receptor is well studied due to its potential role in the treatment of depression and anxiety (Cobos et al., 2008). This receptor has been found throughout the CNS mainly concentrating in the hippocampus, frontal cortex, and olfactory bulb, and may play a role in the development of depression (Hayashi and Su, 2005). DMT is one of the only known endogenous sigma-1 agonists (Kd = 15 μM) but its affinity for sigma-1 receptor is 100-fold lower than that of 5-HT2Ar (Fontanilla et al., 2009). Exogenously administered sigma-1 agonists produce behavioural responses similar to exogenously administered DMT, such as decreased number of entries into the open arms of the elevated plus-maze and decreased immobility in the forced swimming test (Villard et al., 2011; Navarro et al., 2012).

The sigma-1 receptors regulate the secretion of brain-derived BDNF (Fujimoto et al., 2012) and various forms of structural and functional neuronal plasticity (Ruscher et al., 2011). Moreover, the sigma-1 receptor knockout mice exhibited a depressive phenotype (Sabino et al., 2009). Finally, it has been shown recently that DMT can protect human cortical neurons from oxidative stress *via* a sigma-1 receptor-dependent mechanism (Szabó et al., 2021). This may be related to the effect of sigma-1 on the endoplasmic reticulum stress response or to the pro-survival properties of BDNF secretion following sigma-1 stimulation.

Trace amine-associated receptor 1 (TAAR1) has also been suggested as a DMT target (Burchett and Hicks, 2006). While DMT has been shown to activate TAAR1 at 1 μM, the exact EC50 value for DMT remains unknown. DMT can also act as a substrate (rather than an inhibitor) for SERT and vesicular monoamine transporter (Cozzi et al., 2009). DMT can bind to dopamine with a rather low affinity (Ki ≈ 5 μM) compared to ergolins, such as LSD (Ki ≈ 20 nM) (Cameron and Olson, 2018).

### Safety

Like most tryptamine psychedelics, DMT can cause some adverse physical effects, including diarrhoea, nausea, and vomiting (Strassman and Qualls, 1994). Additionally, elevated HR, blood pressure, and rectal temperature have been observed following DMT administration (Dos Santos et al., 2018).

Smoking is the preferred route of DMT administration among recreational users (i.e., non-religious use) rather than ayahuasca use. Those who use DMT recreationally also tend to administer a wide range of other illicit substances, including narcotics, psychostimulants, depressants, cannabis, and alcohol, which confounds any conclusions that can be drawn regarding potential negative health effects of using DMT.

Based on rodent studies, the average lethal dose (LD50) for humans for i.v. and oral DMT administration have been estimated to be approximately 1.6 and 8 mg/kg, respectively (Gable, 2007). Death caused by ayahuasca is quite rare (Dos Santos et al., 2017).

Psychologically, DMT can cause short-term emotional distress and, in some cases, precipitate long-lasting psychosis (Dos Santos et al., 2017). However, the latter is exceptionally rare and tends to be an issue only for people who abuse other drugs, have been previously diagnosed with a mental illness, or have a family history of schizophrenia or mania (Dos Santos et al., 2017). When administered in controlled clinical settings, where participants are carefully screened for factors that could predispose them to long-term adverse psychological effects, both DMT and ayahuasca appear to be safe (McKenna, 2007; Barbosa et al., 2009).

There is a common misconception that serotonergic psychedelics, such as DMT, are addictive and associated with significant health risks. DMT and ayahuasca have been found not to promote compulsive drug-seeking behaviour in humans (McKenna, 2004; Bogenschutz and Johnson, 2016).

Known for the ability to produce hallucinations and delusions, DMT was originally considered an endogenous schizotoxin (Schartner and Timmermann, 2020). There is conflicting evidence as to whether endogenous DMT levels are elevated in psychotic disorders, and research so far has been inconclusive (Jacob and Presti, 2005). While ayahuasca and other psychedelics can precipitate psychosis in predisposed individuals, the rates of psychosis in the União do Vegetal (UDV) are comparable to the general population in Brazil (Dos Santos et al., 2017).

One side effect is vomiting, which results from central vagus nerve amplification due to 5-HT stimulation, and diarrhoea can result from peripheral overstimulation of the gut by 5-HT. With regular use, people may develop some physical tolerance (Crowell, 2004; Browning, 2015). Similar to other serotonergic drugs, DMT triggers increased levels of prolactin and growth hormone. Cortisol blood levels can also be increased following DMT administration (Galvão et al., 2018).

Some studies suggest that DMT may activate peripheral 5-HT2Ar on leukocytes with impacts on cytokine secretion, cell differentiation, and glucocorticoid levels, which may have a modulating or inhibitory impact on the immune system (Frecska et al., 2013). Additionally, DMT can increase the levels of secreted interferon-β and interferon-γ in cultured human natural killer cells and that could be mediated by the sigma-1 receptor (Frecska et al., 2013). Also, through the sigma 1 receptor, DMT and 5-MeO-DMT can reduce the production of several pro-inflammatory cytokines (interleukin-1β, interleukin-6, tumor necrosis factor-α) and chemokine interleukin-8, while they increase the secretion of the anti-inflammatory cytokine interleukin-10 in primary monocyte-derived dendritic cells *in vitro* (Szabo et al., 2014).

DMT causes dose-dependent elevations in pupil size (Dos Santos et al., 2012). The amplitude of the pupillary light reflex was reduced and its latency was increased with DMT use (Dos Santos et al., 2011). Compared to placebo, DMT can cause a statistically significant decrease in body temperature during the first hour, followed by a gradual increase (Dos Santos et al., 2011).

A study showed that a DMT dose of 0.85 mg/kg can cause a maximum increase in diastolic blood pressure (DBP) of approximately 10 mmHg at 15 min and a maximum increase in systolic BP (SBP) of approximately 8 mmHg at 75 min (Grzybowski et al., 2003). With respect to the HR, the maximum increase was approximately 5 beats per minute (BPM) at 60 min. Another study found maximum increases in BP at 40 min, 11 mmHg for SBP and 9 mmHg for DBP (Callaway et al., 1999). The HR at maximal increase was 7 BPM above baseline at 20 min (79 BPM), decreased to a minimum of 7 BPM below baseline at 120 min (65 BPM), then returned to baseline at 240 min (Callaway et al., 1999).

When several studies were analyzed to compare changes in HR, SBP, and DBP brought on by various psychoactive substances, the hemodynamic effects of ayahuasca appeared less hazardous than i.v. DMT, oral alcohol, insufflated cocaine, smoked marijuana, and oral methylenedioxymethamphetamine (Gable, 2007). As with any substance triggering acute hemodynamic changes, some minor adverse cardiac events may occur with the use of ayahuasca, although an increase in values of hemodynamic parameters could be attributed to changes in physical activity or other causes, such as anxiety-provoking stimuli.

The LD50 for DMT is 47 mg/kg i.p. and 32 mg/kg i.v. in mice, which is similar to the IV LD50 for other compounds resembling DMT structurally (psilocin, psilocybin, bufotenin, and 5-MeO-DMT), when administered to rodents (Dargan and Wood, 2013). When comparing the toxicity of various psychoactive drugs, ayahuasca has a safety margin similar to codeine, mescaline, and methadone, with ta lethal dose about 20 times the usual effective dose (Gable, 2004). There are no reports of deaths directly related to ayahuasca use. There are a few reported deaths associated with ayahuasca-like herbal preparations. However, these cases were observed when co-ingested with other substances (Sklerov et al., 2005; Gable, 2007).

### Therapeutic Effects in Mental Health Disorders

Ayahuasca appears to be beneficial in the treatment of addictions, depression and, when used appropriately, carries no risk of abuse or dependence (Morgenstern et al., 1994; Palhano-Fontes et al., 2019). Ayahuasca may enable sustained abstinence from alcohol, barbiturates, sedatives, cocaine, amphetamines, and solvents (Fábregas et al., 2010). Compared to matched controls, regular participants in Brazilian church ayahuasca ceremonies scored significantly lower on the Addictions Severity Index subscales of Alcohol Use and Psychiatric Status, although this may be due to their involvement in a supportive community, or both (Fábregas et al., 2010). Adolescents from a Brazilian ayahuasca-using church also had less recent alcohol use (32.5%) compared to adolescents who had never used ayahuasca (65.1%).

Ayahuasca antiaddictive properties may be determined by a reduced dopamine level in the brain or activity in the mesolimbic dopamine pathway, decreasing the reward associated with the addictive substance (Liester and Prickett, 2012). DMT is a known 5-HT2Ar agonist, and 5-HT2Ar activation can inhibit dopamine release in the mesolimbic, nigrostriatal, and mesocortical pathways. Reduced brain dopamine also fits with the elevated prolactin levels with the ayahuasca use (Callaway et al., 1999). In contrast, 5-HT2Ar antagonists can exhibit reduced dopamine blockade (70%–80% blockade) (Andrade, 2010). Thus, DMT can reduce dopamine levels in reward pathways, which impairs the synaptic plasticity involved in the development and maintenance of an addiction.

Furthermore, a study showed that ayahuasca may be a potential candidate for the treatment of cocaine dependence as it showed a statistically significant reduction in cocaine use compared to tobacco or alcohol use (Thomas et al., 2013). In mice, ayahuasca inhibited some of the early behaviours that are associated with the development of alcohol addiction (Oliveira-Lima et al., 2015).

The alkaloid composition of ayahuasca can vary significantly depending on the preparation of the tisane and the analytical method used to determine its constituents (McKenna et al., 1984). Researchers have focused on the effects of DMT and harmine, although it is still unclear what specific roles they play in the antidepressant and anxiolytic properties of the ayahuasca brew. Harmine not only seems to increase the oral bioavailability of DMT through inhibition of MAO, but it also can have profound effects on mood and anxiety (Ruffell et al., 2020). Furthermore, harmine and other MAO inhibitors have a long history of use as antidepressants in humans. Unlike MAO inhibitors, there have been no clinical studies evaluating the anxiolytic and/or antidepressant effects of DMT administered alone (Ruffell et al., 2020).

A single dose of ayahuasca has been shown to be effective in treating patients with recurrent depression (Osório et al., 2015), and it appears to be relatively safe, as long-term ayahuasca users have not experienced cognitive impairments or increased mental health issues (Bouso et al., 2012). Most of what is known about DMT comes from ayahuasca studies; however, there are several reports detailing the effects of DMT on animal behaviour that potentially can be translated to humans. Recently, DMT has been shown to produce a characteristic antidepressant response in the forced swimming test and has displayed therapeutic efficacy in a behavioural model of PTSD in rodents (Cameron et al., 2018).

The antidepressant and anxiolytic effects of DMT correlate with an increase in dendritic spine density, as well as with an increase in the frequency and amplitude of spontaneous excitatory postsynaptic currents in the medial prefrontal cortex (Ly et al., 2018). Importantly, structural and functional neural plasticity following BDNF signalling and mTOR activation is believed to underlie the antidepressant action of ketamine (Ly et al., 2018). Understanding how DMT and ketamine produce similar cellular and behavioural responses despite binding to disparate receptors is an important area of future research.

In an open-label trial in an inpatient psychiatric unit, a single dose of ayahuasca was found to have rapid-acting anxiolytic and antidepressant effects in patients with recurrent depression (Osório et al., 2015).

A double-blind study showed statistically significant reductions of hopelessness and panic-like parameters using standardized questionnaires, the Beck Hopelessness Scale and the Revised Anxiety Sensitivity Index upon acute ayahuasca administration (Santos et al., 2007). Another study showed that the effect of DMT on trace amine receptors may produce an anxiolytic effect (Carbonaro and Gatch, 2016). Furthermore, it has been suggested that DMT acts similarly to serotonin, and 5-HT2r activation has been shown to alleviate panic symptoms (Santos et al., 2007).

There are many reported psychotherapeutic benefits of ayahuasca, but many studies show that this is only when it is used in specific conditions (Hamill et al., 2019). Ayahuasca is now being considered as a tool to facilitate psychotherapy, dissolving the ego, promoting introspection, and assisting in processes of self-analysis (Metzner, 1998).

## Conclusion and Future Perspectives

The recent research has stimulated renewed interest in classic psychedelics as potential alternatives or adjuvants to the existing pharmacological treatments for mental health disorders. That was driven by the high demand for research and development of novel approaches to treating psychiatric disorders, as well as promising results of existing studies. Although, regulations governing the use of psychoactive substances often limit the ability to undertake scientific investigations on their therapeutic effects. Nevertheless, a remarkable discovery of psychedelic research was the finding that the psychedelic treatment model is based on long-term remission of symptoms after administration of one or two doses. In this review, we have summarized data on serotonergic psychedelics, such as psilocybin, ibogaine, and DMT, their pharmacokinetics, mechanisms of action, safety, and therapeutic effects in a range of psychiatric conditions, including depression, anxiety, PTSD, and SUD, which may have wide applications in clinics. Information about these three psychedelics with pharmacological actions distinct from those of current medications for psychiatric disorders has the potential to advance our understanding of the neurobiological processes and therapeutic outcomes achieved by patients with a variety of psychiatric disorders. However, their full therapeutic potential remains largely unexplored. More studies are required on the safety (potential for abuse), efficacy, common side effects, contraindications, and suitable dose and schedule of these compounds. Moreover, the important question of how to better predict their acute and longer-term response needs to be addressed to help adjust existing treatment models and minimize the potential adverse effects associated with their use as monotherapy. In addition, since there is growing interest in psychedelic-assisted psychotherapy a body of robust evidence for its efficacy and safety must be gathered so that it can be routinely administrated by expert clinicians.
