# Corrigendum: Three naturally-occurring psychedelics and their significance in the treatment of mental health disorders

**DOI:** 10.3389/fphar.2023.1184726

**Published:** 2023-03-28

**Authors:** Nataliya Vorobyeva, Alena A. Kozlova

**Affiliations:** ^1^ Hive Bio Life Sciences Ltd., London, United Kingdom; ^2^ Department of Psychiatry and Psychotherapy, University Hospital Carl Gustav Carus, Technische Universität Dresden, Dresden, Germany

**Keywords:** serotonergic psychedelics, mental health, psilocybin, ibogaine, DMT

In the published article, there was an error in the **Figures**. The figure for Figure 2. An overview of the characteristics of ibogaine was placed as a copy of Figure 3. An overview of the characteristics of DMT. Original incorrect Figure 2 is appear below.

**FIGURE 2:**
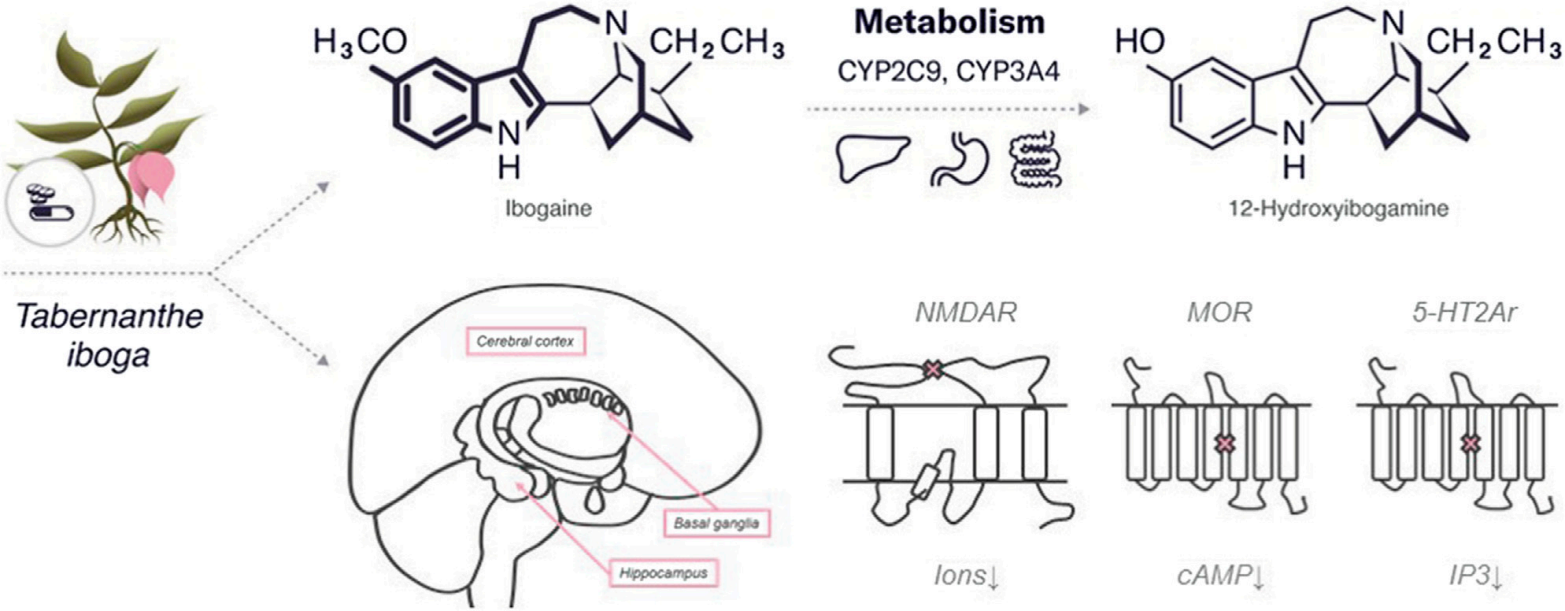
An overview of the characteristics of ibogaine.

The authors apologize for this error and state that this does not change the scientific conclusions of the article in any way. The original article has been updated.

